# Dental Vulcanite Controversy

**Published:** 1874-12

**Authors:** 


### THE DENTAL VULCANITE CONTROVERSY.

The case recently decided in the U. S. Circuit Court at Detroit, involving the validty of the Cummings' patent is one to which the profession has looked with considerable interest, from the fact that great expectations had been entertained in some quarters, at least, as to the results. But just as in other instances, the decision was against the profession.

We were somewhat mistaken in reference to this case, in two particulars at least. There was a general impression that this case would be tried *du novo*—irrespective of those that had gone before. Such however proved not to be the case, for the Judge in giving the descision referring to the former cases, says:

"These are decisions of co-ordinate tribunals of the National Court of the United States; and while it is urged, with great legal ability by the eminent solicitor for the defendants, that these decisions ought not to be and are not conclusive upon this court; yet we feel that such *a series* and unbroken chain of precedents, adjudication and judgments of co-ordinate jurisdictions become obligatory to us. For the purpose at least of uniformity we are constrained to follow, yea, compelled to do so, and in an ordinary case we should decline to enter into the merits, more than to hear arguments, showing that the same or nearly the same points are involved."

Thus we see that from the beginning the court had no intention of hearing the case anew.

In the second place it was positively affirmed that some new points were to be introduced and established in this case. In this particular, as we are informed, there was also failure, nothing new of any importance, whatever, was even presented. Great care should be exercised in all such things, not to excite expectations that will never be realized. We have in this matter however no censure for any one. The profession, throughout, of course, hoped that the best phases were true, and that success in this case would be for them.

The course of the Court in this case, as in some others, seemed to the uninitiated very strange. It is a little difficult to see why the judgments and decisions of one Court, or of several Courts, should be "obligatory" upon another. And why "for the purpose of uniformity this Court was constrained to follow, yea, compelled to do so," we can not understand. Upon this theory all the pleadings, gathering testimony, and indeed all the work done, and money spent was superfluous. The Court had only to look to the decisions that had already been rendered in the same line before, and repeat.

His theory as it seems to us would be a great improvement in judicial affairs, for in the great majority of instances it would only be necessary to find a precedent, and decide by that, and thus end the matter briefly, and at once.

The dentists, those at least who had high expectations, are disappointed, and all wish to know what to do.

We say now, as we have all along said to those who can, abandon the use of rubber in the construction of artificial dentures, and use something else. To those who can not give it up, make the best arrangement you can for its use with the powers that be.

Since this decision it will be useless to resist, for the present at least. The Smith case tried in Portland a few months ago will immediately be taken to the Supreme Court, but a decision can not probably be obtained in less than twelve to eighteen months; and the probability is that the Circuit Courts will grant injunctions whenever asked for.

All these decisions have not in the least changed our opinion as to the invalidity of the Cummings' patent.

